# The Visual Attention and Psychological Responses from Older Customers to Wellness Service Pictures of Hotels

**DOI:** 10.3390/ijerph19031084

**Published:** 2022-01-19

**Authors:** Tsai-Chiao Wang, Ta-Wei Tang, Chia-Liang Tsai

**Affiliations:** 1 Institute of Physical Education, Health & Leisure Studies, National Cheng Kung University, Tainan 701, Taiwan; chiao.ellen@gmail.com; 2 Department of Leisure and Recreation Management, Asia University, Taichung 413, Taiwan; 3 Institute of Innovation and Circular Economy, Asia University, Taichung 413, Taiwan

**Keywords:** yoga, wellness service picture, attention restoration theory, eye-tracking

## Abstract

Understanding the visual attention and psychological responses of consumers to marketing pictures allows hotel managers to design more attractive advertisements. Therefore, the purposes of the present study were to use an eye-tracking analysis to explore whether there were different effects from tourist hotels’ wellness service pictures based on whether they had natural or built clues. The psychological responses with regard to perceived well-being and willingness to pay were also examined. Eighty-five older consumers were recruited. Their eye movement performance while observing marketing pictures with different visual clues and their subsequent psychological responses were measured. It was found that wellness service pictures with natural clues captured more visual attention and induced higher willingness-to-pay perceptions than those with built clues in these older consumers. The present results suggest that marketing pictures with natural clues may create positive visual responses in older customers and further enhance their purchase intention.

## 1. Introduction

The health market segmentation of the hotel industry is gradually expanding [1,2], and the main consumer group of this market is older consumers (age 65 and older) [3]. Compared with young people, older consumers feel that they have limited time [4] and expect to suffer losses in the future [5]. Therefore, they are concerned more with goals related to avoiding loss, such as maintaining body functions and delaying aging. However, hotel researchers still do not understand the impact of health service marketing advertisements provided by hotels on the visual and psychological responses of older consumers who are actively seeking methods to avoid losses.

To obtain a gradual expansion of the wellness market, hotels have started to focus on wellness service images when designing marketing images. Wellness service is defined as the service packages that provide appropriate professional knowledge and individual care to preserve or promote customers’ health [6]. In the hotel field, natural and built servicescapes represent the two ends of the environment design spectrum [7]. Natural servicescapes refer to the use of the natural environment (e.g., mountains, rivers, tropical rain forests, and oceans) to build servicescapes in which consumers can enjoy physical activities [7]. Natural servicescapes can shorten the distance and facilitate the interaction between individuals and natural environments [7,8], allowing consumers to enjoy a relaxing experience. Hotels can use the natural environment as a background for physical activity and encourage consumers to engage in physical activities in the natural environment. Built servicescapes refer to the use of indoor space (e.g., sound, visual design, functional equipment, and atmosphere) to create a better exercise experience [7,9]. Hotels can influence consumer behavior by designing visual images of the physical environment, providing functional equipment, and creating relaxing spaces. Built servicescapes provide attractive designs, pleasant environmental conditions, sufficient activity space, and proper facility functioning to ensure that customers can perform physical activities satisfactorily [9]. To design an effective wellness service image and promote the development of the wellness service market, this research aims to confirm the influence of marketing images based on natural or built servicescapes on the visual attention and psychological responses of older customers in terms of their well-being and willingness to pay.

In order for hotels to design tourist wellness service pictures that can effectively attract older customers, it is necessary to explore how to design the characteristics of the pictures from their perspective. Attention restoration theory argues that natural environments can easily attract the attention of individuals and stimulate them to engage in physical activity in such an environment [7,10]. The interactive relationship between individuals and natural environments can improve the emotional states of individuals, which in turn leads to a number of restorative outcomes [11]. According to previous findings on the attention restoration theory in the fields of marketing [12,13], tourism [7] and environmental psychology [11,14,15], hotel marketing managers can consider designing images that have visual clues related to recovery from mental fatigue, stress reduction, or improved emotional states, thereby improving marketing effectiveness [7].

To accurately investigate the influence of image features on older consumers’ visual and psychological responses, an eye tracking technique was adopted in the current study to record individual eye movement trajectories and analyze visual attention [7,16]. Previous researchers have used this method to conduct choice experiments, where it was found that this technology makes it possible to obtain real-time eye movement information that expands the scope of information obtained from analyzing individual preferences [17,18]. For example, Wang et al. (2019) [7] found that images of natural servicescapes may attract more visual attention and arouse higher restorative perceptions among potential customers than built servicescapes.

The purposes of this study were thus to explore the potential divergent effects of hotel wellness service pictures with natural or built clues on visual attention, perceived well-being, and willingness to pay in older consumers. The findings will not only provide insight into the academic development of attention restoration theory in the tourism and hospitality field, but will also contribute to the ability of hotel marketing managers to develop effective tourism service advertisements.

### 1.1. Wellness Service—Yoga

Many countries are beginning to face the challenge of an aging population. The ageing of the population has gradually increased the age of consumption in the hospitality industry, which in turn has created a new market segment. Compared to other age groups, the elderly population is a group of people with higher amounts of liquid capital and more travel experience [19,20]. Older adults are motivated to engage in tourism because it contributes to keeping them healthy and relaxed [21,22].

Because well-being is a serious concern of older consumers, wellness services are the most attractive leisure option for the rapidly ageing population. Wellness services allow tourist hotels in the competitive tourism market to increase their operating income by enriching the choices of tourism products [23]. As a result, many hotels have started to use wellness as a part of their business strategies. The wellness service market differs from that of the traditional hotel market. Up until the present, research on wellness services has been rare, leading to a lack of understanding on the part of hotel researchers and managers of this potential market segment [20].

Yoga is suitable as a physical activity for older adults and yields both physical and psychological benefits. Many studies have also confirmed that yoga can effectively improve emotions in older adults (e.g., reducing depression and stress and improving mood) as well as cognitive functions (e.g., attention, memory, and resiliency) [23]. Thus, previous research in the field of well-being tourism has reported that older tourists are the target market for yoga tourism marketing. For example, Ali-Knight and Ensor (2017) [1] explored yoga tourist profiles and found that seniors prefer this type of physical activity.

### 1.2. Older Consumer Responses after Observing Wellness Service Pictures

Tourists always expect to recover from mental fatigue and reduce stress during a vacation [7], so they pay special attention to wellness services that meet their needs. If the marketing messages provided by the hotel meets consumers’ expectations for recovering from mental fatigue and relieving stress, this will affect their preferences, behavioral intentions, and choice decisions. Attention restoration theory is a cognitive theory that focuses on recovery from mental fatigue [14]. Spending time in an environment with a recovery potential has been regarded as a means of restoring life energy [14,24]. In the field of marketing and tourism research, such theories are considered to be an effective tool that can be used to understand and design marketing images and servicescapes that convey a restorative quality and influence behavioral decisions [7].

According to the attention restoration theory, there are three key responses of older consumers after observing the wellness service images: visual attention [7,15,25], perception [26,27,28], and behavioral responses [29,30]. Visual attention is the first response, while customers look at tourism service images (or videos, products, etc.). It reflects the positioning of wellness service images that older consumers are interested in. The second reaction is perception, which is an assessment of the subjective well-being of older consumers (e.g., the degree of well-being they perceive after participating in a yoga program). The third reaction is a behavioral response, which represents the behavioral intention of older consumers after they observe wellness service images.

### 1.3. Eye Tracking and Visual Attention

Visual attention is defined as the allocation of information processing abilities [31] and it is used to describe an individual’s selective observation activities toward images (or videos, products etc.) [18,32]. Researchers can understand customers’ visual behavior through analyzing their visual focus when they look at hotel advertising images using eye tracking technology. Eye tracking systems are a form of sensor technology that can be used to objectively collect data related to a customer’s internal responses and that avoid the common method variance generated by collecting subjective measures through questionnaires [17].

Attention is a selective mechanism for determining the extent to which a customer is concerned about a specific stimulus [33,34]. Attention will directly influence product selection [35]. Customers do not treat all marketing images the same way [32]; rather, they assign more visual attention to features they are interested in. Customer attention can thus be assessed by observing eye movements [35]. In the marketing field, the eye tracking technology used to capture individual eye movements has been used to assess customer attention toward advertising [36]. It has been demonstrated to be an effective tool for assessing the appeal of images [18]. Accordingly, eye movement is thought to be an effective measure of visual attention [37], and can also reflect the content to which viewer attention is directed [38].

### 1.4. The Relationship between Environmental Clues in Images and Visual Attention

Visual attention is a selective process that assigns limited perceptual capabilities related to specific characteristics of wellness service images, while ignoring others [39]. Through these processes, older consumers transfer their attention from the wellness service images as a whole to the part they are most interested in [32].

The results of a green exercise study showed that older consumers prefer to be close to nature rather than in indoor sports environments, because nature can satisfy their pursuit of natural experiences and convenience motivations [40]. Although indoor sports environments adopt a closed space design and provide individuals with full-length mirrors to exercise in front of them, so they can observe the physical changes resulting from physical activity, built images are less likely to attract observers’ visual attention and are less able to provide psychological relaxation benefits to consumers [7]. Wang and Sparks (2016) [18] found that natural images, as compared to built images, attract more frequent visual attention from customers. In addition, based on the attention restoration theory, as mentioned above, Calogiuri et al. (2015) [40] found that when individuals exercise in a natural environment, their attention will shift to the environment, rather than to their inner fatigue, leading to a decrease in perceived fatigue. In addition, consumers may have greater visual responses to pictures with natural images due to those images providing more psychological benefits [7] than pictures with built images. Therefore, compared to pictures that provide built clues, wellness service pictures that present clues related to connecting with the natural environment may be more attractive to older adults. Based on the previous discussion, the following hypothesis is proposed:

**Hypothesis** **1 (H1).** *Older consumers will direct more visual attention towards images with visual clues depicting nature than to wellness service images with built clues*.

### 1.5. The Relationship between Environmental Clues in Images and Well-Being

The natural environment can be used as a wellness resource that promotes physical and psychological well-being [26,41]. In particular, untouched surroundings are the physical environment necessary to create a transformational well-being experience [42]. When individuals are surrounded by nature, they have opportunities to reflect on the relationship between humans and the environment [14]. In addition, the natural environment also helps individuals recover from mental and physical trauma. According to attention restoration theory, compared to physical activity in a built environment, physical activity in a natural environment allows older consumers to experience both the beneficial effects derived from nature and from physical activity [10]. For example, using middle-aged to elderly adults as participants, the results of Astell-Burt et al. (2013) [27] confirmed that physically active adults living in a green environment experience a lower risk of psychological distress.

Images can convey emotional messages, in turn allowing observers to imagine what the environment provides. When individuals look at a tourism image, they can imagine themselves in the servicescape depicted in the image and can imagine experiences in that environment [43]. When potential consumers see beautiful pictures of natural environments associated with a tourist hotel, their fascination for nature is aroused, and therefore positive emotions toward the hotel are increased [7]. Previous research has reported that compared with wellness service images that provide clues related to an indoor sports environment, pictures with clues about nature provide individuals with more effects related to reducing fatigue and increasing relaxation and stress relief [26]. In addition, Rosenbaum et al. (2016) [44] confirmed that shopping centers can promote personal and social well-being by providing shoppers with a restorative servicescape. Their research not only found that shopping centers that incorporate green elements into the retail area promote wellness, but also found that shoppers who sense recovery quality in such environments develop good attitudes and exhibit positive behavior toward shopping centers. Accordingly, older consumers who observe wellness service images with clues suggesting a natural environment will tend to have a higher sense of well-being, where these images can evoke positive emotions and even enhance expectations of an improved sense of mental and physical well-being when practicing yoga in natural spaces. Based on the previous discussion, the following hypothesis is proposed.

**Hypothesis** **2 (H2).** *Compared to observing wellness service images with built visual clues, older consumers will perceive a higher sense of well-being from wellness service images with natural visual clues*.

### 1.6. The Relationship between Environmental Images and Willingness to Pay

Evaluations of marketing effectiveness using wellness service images are usually accompanied by the assessment of customer willingness to participate in wellness services and pay fees. A consumer’s willingness to pay for a service is defined as the maximum price a consumer is willing to pay for a given service [45,46]. Thus, consumers may have a higher willingness to pay for wellness services that they prefer. Customers are willing to pay a relatively high price to purchase high-level service quality that maximizes consumption value [47]. When older adults recognize that wellness service images with nature clues (e.g., learning yoga activities in the natural environment) can satisfy their pursuit of wellness, their willingness to pay higher fees will increase.

The attention restoration theory argues that exercise in the natural environment not only reduces physical and mental fatigue in older adults [29], but also enhances the effectiveness of physical activity [28,48]. Wellness service images that provide nature clues make it possible for consumers to recognize that a tourist hotel can provide opportunities for them to interact with nature and simultaneously engage in physical activity. A high-level restorative experience can lead consumers to spend more time and money to enjoy a memorable consumer experience [49]. For example, Rosenbaum, Otalora, and Ramírez (2016) [44] found that consumers’ planned shopping expenditures increased after a highly restorative experience. Thus, consumers are willing to pay more for shopping in a highly restorative retail service environment rather than in a less restorative environment [49]. Therefore, wellness service images with clues suggesting an experience in nature are expected to have a higher impact on older adults’ willingness to pay more than those with indoor sports clues. Based on the previous discussion, the following hypothesis is proposed.

**Hypothesis** **3 (H3).** *Compared with observing wellness service images with built clues, older consumers observing images with nature clues will be willing pay more for services*.

## 2. Methods

### 2.1. Participants

Older adults (age 65 and older) were the source of the study sample. The researchers contacted participants in the database of the Taichung Senior Citizens Association in Taiwan through flyers or personal visits, and invited them to participate in the experiment.

The criteria for selecting participants were as follows: (1) Individuals were over the age of 50, (2) the participants had normal vision in both eyes (more than 0.8 after correction) and had no major eye diseases (e.g., macular lesions), and (3) individuals could not physically move were excluded. This decision was made so as to ensure that the participants would not be discouraged from practicing yoga owing to physical limitations.

To effectively control the experiment, 100 candidates were contacted and agreed to participate in this study. However, 15 participants with binocular vision not reaching 0.8 or more and major eye and monocular data of 0 were excluded. A total of 85 valid participants were enrolled in this study, with an effective sampling rate of 85%. The Mini-Mental State Examination (MMSE) [50] is the well-established measure tool for cognition. An MMSE score above 26 was used to classify adults as not being cognitively impaired [50]. The average age of the participants was 66.54 years old, and the average MMSE score was 27.80. Of all participants, 42 were males and 43 were females;

A G*Power analysis was performed to determine whether the sample size was sufficient to verify the research hypothesis [51]. The results of the G*Power analysis showed that the study had an 80% power to detect small effects (d = 0.27) at the 5% level of visual attention significance. The G*Power analysis results thus indicated that the sample size of this study was sufficient to test the hypotheses.

### 2.2. Selection of Stimuli

**Figure 1:**
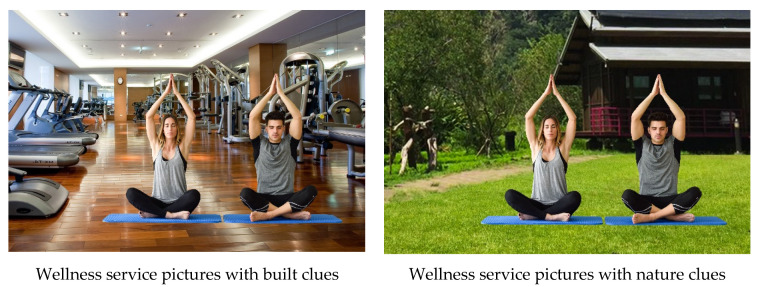
Two samples of the experimental pictures (portraits adopted from https://www.freepik.com/free-photo/health-beautiful-female-body-peace_1057237.htm#page=8&query=gym%20background&position=8, accessed date 1 December 2021).

To select the images and minimize the confounding factors between them, the following processes were used in the present study [7]. First, the pictures used as the stimuli were designed with the same yoga posture, as yoga is a low-intensity exercise mode and is suitable for promoting the wellness of older individuals and preventing diseases [52]. Second, the backgrounds of the pictures were designed with both nature clues and built clues, with the nature clues referring to the use of natural environments (e.g., grasslands, oceans, mountains, and other natural landscapes) and the built clues referring to indoor-exercise-space materials (e.g., floors, carpets, mirrors, and cement walls). Third, three experts in the physical activity field were invited to remove photos with low brightness and insufficient clarity, or those that were considered unsuitable for older adults, with 36 pictures being rejected. Each picture had a single coach performing a yoga posture that can be performed by any participant in the sample. At this step, photos with specific customer characteristics were also rejected. Finally, based on the consensus of the experts, 16 pictures were selected, with eight pictures having nature clues and eight having built clues. Samples of the experimental pictures are shown in Figure 1.

### 2.3. Experimental Procedure

The participants were asked to pretend that they were watching online marketing advertisements provided by hotels in the form of wellness service pictures, after which their vision, attitude, and willingness to pay reactions after watching these advertisements were explored. The participants were told that they had sufficient budget to purchase exercise courses. This was intended to eliminate budgetary constraints related to purchasing exercise services. The price of each exercise plan was not included in the advertisement, as this could have interfered with the service selection process.

Before the experiment, participants were asked to state their health status and to confirm that they had no mental illness or cognitive impairments, as assessed by the MMSE (i.e., scores > 24), and had normal or corrected vision greater than 0.8 [53]. All participants signed an informed consent form approved by the Human Research Ethics Committee of the National Cheng Kung University in Taiwan. The participants were then randomly assigned to either the natural environment group (n = 43) or the built environment group (n = 42). Each picture among the eight pictures was randomly selected for each group and was exhibited for 12 s to participants in an acoustically shielded room with dimmed lights (Wang et al., 2108a; Wang and Sparks, 2016). After each picture was displayed for 12 s, the next picture was displayed.

An eye tracker was used to record the eye movement trajectory of the participants while observing the experiment pictures. Before the eye tracker began recording eye movement data, a nine-point calibration procedure was performed. After collecting the eye movement data, the participants were asked to complete questionnaires providing information about their sense of perceived well-being and their willingness to pay to participate in the yoga program after observing the picture. The participants’ gender, age, and travel frequency from the previous year were also collected. The entire experiment took approximately 1 h.

### 2.4. Instruments

An eye tracker (sampling rate 60 Hz) (Tobii X2-30, Danderyd, Sweden) placed in front of a 24-inch widescreen TFT monitor (1920 × 1080 pixels) was used to present the stimuli and to capture the eye movement information from the participants. Each participant was 60 cm away from the screen monitor.

### 2.5. Measurement

Visual Attention. Eye tracking technology visualizes visual attention in the form of a gaze plot, allowing researchers to explore visual attention. The key indicators used to assess individual visual attention were the fixation count and the fixation time [37,54]. The fixation count is the number of times the individual interacts with the stimuli, where a greater count of fixation indicates that the participant finds the information to be more attractive [7,36]. The fixation time indicates the processing time for the individual to watch the stimuli, where a longer fixation time indicates that the individual spent more time examining the information or the relationships between the internal and external representations [34,55]. Therefore, images with a longer fixation time were observed, indicating that the participants’ visual attention was attracted more during that time [7,36]. Individual visual attention typically lasts from 200 ms to 500 ms. As neuroscience visuo-cognitive research usually uses a threshold of 200 ms [56,57], values below 200 ms were removed [18].

Perceived well-being. The scale developed by Tseng and Shen (2014) [58] was used to measure perceived well-being, and seven items were used. The participants were asked to express their perspectives after observing the wellness service images, including what degree of physical wellness and mental wellness they would feel if they participated in such a wellness service. These measures consisted of items with response options ranging from 1 (strongly disagree) to 7 (strongly agree).

Willingness to pay. The psychological response of willingness to pay was measured using one item. The researcher asked the participants to respond to the following statement: To assist a hotel marketing manager of a tourist hotel in the pricing of wellness services, please state the maximum price you would be willing to pay to participate in the wellness services provided by the tourist hotel in the marketing image. The participants provided the maximum amount they were willing to pay in the form of an open-ended response [59].

### 2.6. Statistical Analysis

The data were analyzed using a t-test and descriptive statistics with SPSS version 21.0. The descriptive statistics were presented as means and percentages for willingness to pay in different categories. The t-test for the dependent variables was used to examine the differences in the visual attention, perceived well-being, and willingness to pay.

## 3. Results

As indicated in Table 1, the average time spent by the participants observing wellness service images with nature clues was higher than that spent observing images with built clues (t = 2.10, *p* = 0.037). Therefore, the yoga pictures with natural characteristics attracted more visual attention from the participants. Thus, the results supported Hypothesis 1.

There were no significant differences found in the observations of pictures with natural and built clues in terms of perceived well-being (See Table 1). Therefore, Hypothesis 2 was not supported.

As shown in Table 2, the natural and built characteristics of wellness service images had different effects on the psychological response of willingness to pay in participants. Compared with yoga images with built characteristics, yoga pictures with natural characteristics induced more potential to evoke willingness to pay (t = 2.05, *p* = 0.023). Thus, this result supported Hypothesis 3.

Furthermore, as indicated in Table 3, in the nature picture group, the price range that participants were willing to pay ranged from US $10.00 to US $30.00, with the average amount being US $17.10. In terms of the percentage, participants who were willing to pay less than US $10.00 accounted for 47% of all participants, those between US $11.00 and US $20.00 accounted for 18%, and those between US $21.00 and US $30.00 accounted for 35%.

In the built picture group, the average price the participants were willing to pay was US $14.80. Participants who were willing to pay less than US $10.00 accounted for 49% of all participants, those between US $11.00 and US $20.00 accounted for 29%, and those between US $21.00 and US $30.00 accounted for 22%. The *t*-test results showed that participants who examined the natural images had a higher level of willingness to pay than those who watched the built pictures (nature vs. built: US $17.10 vs. US $14.80, t = 2.53, *p* = 0.0023; see Table 3).

## 4. Discussion

Images comprise an important medium for transmitting marketing messages and are also an important mode of evaluation for individuals prior to making accommodation decisions. A strategy using natural images in wellness services seems to be a feasible way to attract the visual attention of older consumers and to facilitate their purchase intentions. By using the power of market-oriented economies, service providers can create new business and green service models, integrating natural images and service differentiation strategies into their sports firm’s overall marketing programs. The four major findings of the present study are as follows.

First, using the eye-tracking technology, it was possible to accurately capture the older consumers’ visual preferences for wellness service pictures and to understand their implicit physical activity decision-making process. It was found that older customers showed a longer fixation time and larger fixation frequencies when observing wellness service pictures with nature clues as compared to those with built clues. Previous research has also reported similar results when a similar group observed wellness service pictures with nature clues as compared to indoor built clues [30]. Indeed, when older consumers look at wellness service pictures for a limited period of time, they selectively prefer those with nature clues [7]. It is likely that wellness activities performed in the natural environment will strengthen the attention restoration effect and facilitate a higher restoration quality, in turn causing consumers to feel more relaxed [7,60]. This concurs with Li et al.’s (2016) study exploring the visually attractive characteristics of tourism and leisure sports marketing images. They found that images with nature clues captured visual attention more easily. Likewise, Wang et al. (2019) [7] used an eye-tracking analysis and found that marketing images with natural landscapes and artistic components yielded better advertisement effectiveness than those with built environments and artistic components in customers at an average age of approximately 55 years. Considered jointly, the previous and present results suggest that natural landscapes seem to induce more visual attraction in this group.

Second, the results of the present study indicated that observing wellness service pictures with nature clues compared with built clues did not lead to higher perceptions of well-being. This result was somewhat inconsistent with the findings of Mitchell (2013) [48], who found that individuals engaging in physical activity in nature relative to other environments exhibited better mental health. Wang et al. (2020) [60] also found that individuals exercising in an environment with natural landscapes exhibited higher levels of stress-relief, restorative quality, and personal satisfaction compared to those exercising in an environment with a virtual abstract painting. Accordingly, considering these contradictory results, we are cautious in suggesting that perceived well-being is not truly elicited through observation of pictures with nature clues. Previous research confirmed that the restorative qualities obtained from visiting nature will enhance well-being through psychological and physical benefits [61]. However, observing pictures does not directly induce well-being [62]. Without actually visiting these natural settings, it is difficult to perceive the benefits (psychological or physical benefits) of interacting with the natural environment [62]. Furthermore, the participants who observe the pictures may only have perceived lower restorative qualities because they have not actually visited these natural environments, or the selected pictures did not provide higher perceptual restorative qualities.

Third, to the author’s knowledge, this is the first study to investigate older consumers’ willingness to pay after observing wellness service pictures. It was found in the current study that participants in the nature group were willing to pay an average of US $17.10 to enjoy the yoga activity, which is higher than what the participants in the built group were willing to pay. However, it is worth noting that the nature group showed two extremes (i.e., less than US $10.00 and between US $21.00 and $30.00) in terms of willingness to pay, suggesting that there are two distinct market segments when hotels provide a wellness service in a natural environment. Individuals with different characteristics will interpret and place different values on the same thing [36,54]. Although this conjecture is somewhat speculative, it provides a basis for future research.

The findings show that wellness service pictures with nature clues not only elicited higher levels of visual attention in the older consumers under consideration, but also led to willingness to pay. The empirical results suggest that visual clues in hotel marketing pictures can influence customers’ visual behavior and assessments of willingness to pay. In particular, images of natural servicescapes may attract more visual attention than those of built servicescapes, and natural servicescapes may also signal higher restorative quality to older potential customers. This finding was consistent with the arguments of attention restoration theory, suggesting that a natural environment can reduce mental fatigue in older consumers and provide incentives for physical activity. Therefore, if older consumers believe that they can learn yoga in an outdoor space with green trees, plants, and grass, this may induce motivation toward willingness to pay. As natural environments provide restorative resources, relaxation, leisure, social, and health benefits to individuals, they may be willing to pay more for experiences in such environments [63]. This study expands the concepts of attention restoration theory to the willingness to pay research field, reflecting that customer’s positive responses to psychological/affective restoration may interact with purchase intention. Therefore, if hotels can satisfy customers’ wellness needs or provide stress-relieving activities, they will potentially obtain a competitive advantage [7,19].

### Research Limitations and Future Research Suggestions

In this study, eye-motion performance, perceived well-being, and willingness to pay were used to investigate the psychological responses of older consumers to wellness service (e.g., yoga) images. Whether they would actually participant in such a physical activity was not investigated. As a result, we could not clearly confirm the actual contribution of the wellness service pictures in term of whether they actually caused the older consumers to engage in yoga during their hotel stay. It is suggested that future studies may further explore the characteristics of images that enable older consumers to feel a sense of well-being and how these images influence subsequent physical activity behavior and outcomes. In addition, using a longitudinal study to explore the potential relationships between different types of perceived well-being obtained via observing wellness service images (e.g., yoga) with nature clues and via practicing the similar physical activity in a hotel’s outdoor natural environment is warranted.

## 5. Conclusions

Tourists might choose a hotel based on their perception of its ability to release stress and promote wellness. The present findings show that wellness service strategies incorporating natural environments appear to make consumers more willing to use the service and willing to pay higher prices to enjoy it. Thus, hotels wanting to increase their profit can provide activities in the natural environment (e.g., allowing customers to participate in a yoga activity via interaction with the outdoor natural environment) to provide psychological and mental health benefits to their older guests.

## Figures and Tables

**Table 1:** Effects of image characteristics on older consumers’ visual attention.

Visual Attention		Image Characteristics	
Image characteristics	Eye-movement	Nature clues	Built clues
Fixation time	Seconds	11.69 ± 0.36	11.29 ± 1.17
	t	2.10 *	
	*p*	0.037	
Fixation count	Frequencies	585.74 ± 147.55	524.86 ± 179.19
	t	1.78	
	*p*	0.143	
Well-being		4.28 ± 0.49	4.19 ± 0.43
	t	1.04	
	*p*	0.298	

* *p* < 0.05.

**Table 2:** Effects of image characteristics on willingness to pay in older consumers.

Image Characteristics	Means	t-Value	*p*
Natural environment	513.26 ± 130.45	3.85 *	0.016
Built environment	443 ± 93.78		

* *p* < 0.05.

**Table 3:** Comparison of the amount older customers were willing to pay.

Range of Willingness to Pay	Characteristics of Wellness Service Images	
	Nature clues (%)	Built clues (%)
Less than 300 (NT dollars)	20; 47%	21; 49%
301–600 (NT dollars)	8; 18%	12; 29%
601–900 (NT dollars)	15; 35%	9; 22%
Average (NT dollars)	$523 (approximate US $17.10)	$443 (approximately US $14.80)

## Data Availability

No data sharing is applicable to the current article.
